# PROLONGATION OF HUMAN LIFESPAN BY IMMATURE PEAR EXTRACT MEDIATED SIRTUIN-RELATED GENE EXPRESSION

**DOI:** 10.1093/geroni/igz038.365

**Published:** 2019-11-08

**Authors:** Akira Ogita, Wakae Murata, Marina Hasegawa, Ken Yamauchi, Akiko Sakai, Yoshihiro Yamaguchi, Toshio Tanaka, Ken-ichi Fujita

**Affiliations:** 1 Graduate School of Sciences, Osaka City University., Osaka, Japan; 2 Department of Materials Science, National Institute of Technology, Yonago College., Yonago, Tottori, Japan; 3 Institute of Physical Education, Keio University, Kanagawa, Japan; 4 Research Center for Urban Health and Sports, Osaka City University., Osaka, Japan

## Abstract

Demographics of the world are changing rapidly with older populations growing at an unprecedented rate. Cellular senescence, a decline of cellular function due to aging, causes gradual loss of physiological functions. Several cellular senescence-related chronic diseases, such as metabolic syndrome, cardiovascular disease, cancer, osteoporosis, diabetes, and hypertension, negatively affect the quality of human life. Intervention in the cellular senescence process may reduce these incidences and slow the progression of age-related diseases, while contributing to the longevity of healthy human lifespans. Saccharomyces cerevisiae, the budding yeast, is a simple model system that can provide significant insights into the human genetics and molecular biology of senescence and is considered suitable as a cellular model for research on mammalian cells. The aim of our study was to investigate the anti-aging effects of immature pear fruit extract (IPE) on yeast cells and its possible application to extend healthy lifespan in humans. Anti-aging effects of IPE were investigated using a chronological lifespan assay on S. cerevisiae cells. The chronological lifespan of the yeast treated with IPE at 1% (v/v) was significantly extended than that of untreated cells (p < 0.05). The expression of sirtuin-related genes, which regulate cellular senescence, was examined by reverse transcription-polymerase chain reaction and found to be significantly increased following IPE treatment. These results suggest that sirtuin-related genes have important roles in IPE-regulated lifespan extension, which provides a mechanism by which IPE could affect mammalian cells and potentially extend healthy human lifespans.

